# Adolescents' experience of receiving internet-delivered cognitive therapy for social anxiety disorder

**DOI:** 10.1016/j.invent.2023.100664

**Published:** 2023-09-07

**Authors:** Eleanor Leigh, Rosie Nicol-Harper, Mariana Travlou, David M. Clark

**Affiliations:** a Department of Experimental Psychology, University of Oxford, Oxford Health NHS Foundation Trust, UK; b Department of Experimental Psychology, University of Oxford, UK; c Department of Experimental Psychology, University of Oxford, UK

**Keywords:** Adolescent, Social anxiety, Cognitive therapy, CBT, Qualitative

## Abstract

**Background:**

Social anxiety disorder (SAD) is a debilitating condition that usually begins in adolescence. We recently demonstrated preliminary efficacy of an internet-delivered therapist-assisted version of Cognitive Therapy for Social Anxiety Disorder (SAD) for adolescents called Online Social anxiety Cognitive therapy for Adolescents (OSCA). Here we report on the helpfulness, support, and overall acceptability of OSCA from the perspective of trial participants.

**Methods:**

Participants were 17 young people aged 15–18 years who had participated in a trial of OSCA. Post-treatment, participants completed an online treatment acceptability questionnaire and took part in a semi-structured interview to gain an understanding of their experience of OSCA.

**Results:**

Overall, there was a very high rate of treatment satisfaction. Core aspects of the treatment were viewed as most helpful, including behavioural experiments even though participants found them challenging. Participants found the online nature of the treatment helpful, allowing for easier communication with the therapist, regular encouragement from the therapist, and the ability to go back to their treatment and view their progress. Challenges were, for some, the quantity of content and practical issues around scheduling the short weekly calls with their therapist.

**Conclusions:**

This study suggests that young people felt helped and supported by OSCA.

## Highlights

• Treatment satisfaction was high overall.
• Therapist communication and support was rated as important.
• Core aspects of treatment were considered challenging but most helpful.
• Flexibility offered by online delivery was viewed positively.

## 1 Introduction

Social Anxiety Disorder (SAD) is characterised by a persistent fear of embarrassment or humiliation by others. It is the most common anxiety disorder with a lifetime prevalence of 7 % (Fehm et al., 2005) and it usually starts in late childhood or adolescence (Burstein et al., 2011).

Cognitive Therapy for SAD (CT-SAD) was designed to target the maintenance processes specified in the cognitive model of Clark and Wells (1995), namely self-focused attention, negative self-imagery, and safety behaviours. In adults, it has been shown to be superior to a range of other active treatments (Mayo-Wilson et al., 2014) and it is one of the first-line NICE recommended treatments (National Institute for Health and Care Excellence, 2013). There is a small but growing evidence base showing that CT-SAD can also be effective in the treatment of youth SAD (Ingul et al., 2014; Leigh and Clark, 2016; Leigh and Clark, 2023; Melfsen et al., 2011). For example, in a Norwegian randomised controlled trial with 57 adolescents, CT-SAD was associated with large effect sizes compared to both waitlist and attention control conditions (Ingul et al., 2014).

However, there are considerable barriers that make it difficult for young people to access face-to-face-to-face therapies such as CT-SAD. These include limited availability of specialist staff, high thresholds for referral to Child and Adolescent Mental Health Services, structural barriers to accessing help including travel costs, and stigma related to attending mental health facilities (Radez et al., 2020). Furthermore, compared to adults, young people are less likely to seek traditionally delivered treatment (Radez et al., 2020). One way to potentially overcome some of these barriers is by offering treatment via the internet, which allows for easier access to treatment at a convenient, flexible time and place. Young people interact with the online environment in all aspects of their lives (Lenhart et al., 2010) and so receiving treatment this way may be especially appealing to this cohort. Accessing a treatment online may provide a greater sense of confidentiality and reduced embarrassment (Sweeney et al., 2019) which will be especially important for adolescents with social anxiety for whom feelings of shame and embarrassment are typical (Olfson et al., 2000).

A therapist-assisted internet version of Cognitive therapy for SAD (iCT-SAD) for adults has recently been shown to have comparable effects to face-to-face therapy (Clark et al., 2022). Following consultation with young people, iCT-SAD was adapted for use with adolescents. The resulting treatment is known as Online Social anxiety Cognitive therapy for Adolescents (OSCA; Leigh and Clark (2019)). While using OSCA, the young person has access to a bespoke, secure website and works through core modules designed to target maintenance processes, with additional modules tailored to specific fears (e.g., blushing, feeling boring) and difficulties (e.g., depression). Treatment spans 14 weeks with weekly 20-minute therapist phone calls and regular messaging via SMS and the messaging function of the program.

Preliminary efficacy of OSCA was examined in a randomised control trial (RCT) with 43 young people (Leigh and Clark, 2023). Compared to waitlist, OSCA was associated with large effect sizes on all outcome measures. At post-assessment, 77 % of participants had lost their diagnosis of SAD after OSCA compared to 14 % after waitlist. Findings from the trial suggest OSCA holds promise as an effective, accessible treatment for adolescent SAD. As well as testing the clinical effectiveness of any new treatment it is important to understand users' experience and whether it is acceptable to them. An understanding of young peoples' perceived barriers to initial engagement or continuing motivation alongside any features which are seen as especially helpful can inform further refinement.

Therefore, the aim of the current study was to examine satisfaction and experience of using an internet-delivered therapist-assisted Cognitive Therapy for adolescent social anxiety disorder (OSCA) amongst participants who had received the treatment as part of a RCT.

## 2 Method

### 2.1 Ethical approval & consent-assent procedures

Ethical approval was granted by the University of Oxford Medical Sciences Division Research Ethics Committee (R60464/RE001). The trial was conducted in accordance with the principles of Good Clinical Practice and the Declaration of Helsinki. Participants aged 14 to 15 provided informed assent with informed consent from their primary caregiver; participants aged 16–18 provided informed consent.

### 2.2 Participants

Participants taking part in a randomised controlled trial (RCT) comparing therapist-assisted OSCA to waitlist (Leigh and Clark, 2023) were invited to participate in a post-treatment interview. All young people who participated in the trial were offered treatment (either immediately or after the wait period). One therapist provided support to all participants in the RCT. We invited (the first) 20 people who completed treatment (either delayed or immediate) to participate. This number was selected to provide a broad, unselected sample to maximize possible responses and represents 47 % of trial participants (Green and Thorogood, 2004). Seventeen (85 %) young people agreed and three declined. Participants in this study (*n* = 17) were all female (as opposed to 91 % female in the full trial sample). Mean age was 16.67, sd = 0.97 (median = 17, and range = 15–18).

### 2.3 Intervention

Detailed information is available in the protocol for the RCT (Leigh and Clark, 2019) and in the findings of the trial (Leigh and Clark, 2023). OSCA takes 14 weeks. Users work through eight core modules initially. Then, up to 16 additional modules focusing on particular fears or problems can be released to individualise the programme for each user (see Supplementary Material for a full list of modules). The programme includes a secure video conferencing facility with recording functionality to support online delivery of two core CT-SAD procedures: the self-focused attention and safety behaviour experiment and video feedback. Adolescents can logon whenever they like. During the 14 weeks, users have weekly 20-min phone calls with their therapist and they also receive regular encouragement and support via secure messaging within the programme and SMS texts. Therapist support is used to provide encouragement to engage with OSCA, to help deepen learning of the ideas covered in the programme, and to provide reminders to engage in therapy tasks.

### 2.4 Measures

#### 2.4.1 Treatment acceptability questionnaire

Participants completed a 22-item self-report treatment acceptability questionnaire online that was developed for our internet programmes (see Supplementary Information section for questions). Participants were asked to rate the helpfulness of the website features, the different communication methods, and communication content. Questions were rated on a Likert scale ranging from 0 (not helpful at all) to 5 (extremely helpful). Another item assessed satisfaction with the frequency of therapist contact on a scale ranging from 1 (too little contact) to 5 (too much contact), with 3 indicating ‘just the right amount’. In addition, participants could provide a free text response to the question “What kind of things did your therapist say that you found particularly helpful?”. The questionnaire was completed prior to the interview.

#### 2.4.2 Interview

The semi-structured interview was conducted by a graduate researcher who was not otherwise known to participants. It took place approximately three weeks post treatment, either on school premises or via video link. The interview included 9 questions (See Table 1). They were all open-ended questions, with follow-up prompts as needed. The first two questions also included a Likert scale measurement i.e., “Overall, how satisfied were you with your treatment?” ranging from 0 (Not satisfied at all) to 5 (Extremely satisfied), and “How supported or alone did you feel during the course of treatment?” ranging from 0 (Alone) to 5 (Very well supported). These ratings were included to help scaffold initial responses, in recognition of the age of the participants and their experience of social anxiety.

**Table 1:** Semi-structured interview questions.

1. Overall, how satisfied were you with your treatment?
2. How supported or alone did you feel during the course of treatment?
3. In general, what were the things that you did during treatment that you found particularly helpful?
4. Were there things that you did during treatment that you found less helpful? If so, what were they?
5. Were there any things that your therapist did or said that you found particularly helpful?
6. Did your therapist do or say anything that you didn't find helpful?
7. Did you have any problems in motivating yourself during treatment? How did you manage to keep yourself motivated and stay in treatment?
8. Is there anything that the therapist, programme, or school could have done to make it easier?
9. Were your parents involved in your treatment? Would you have liked your parents to be more involved?”

## 3 Analysis

Descriptive statistics were calculated for the questionnaire. The semi-structured interviews were audio-recorded and transcribed. Transcripts were read by three members of the research team. Thematic analysis (Boyatzis, 1998) was undertaken. This approach is not associated with a particular theoretical framework (Braun and Clarke, 2006) and was chosen to allow for maximum flexibility to capture the full range of responses from this specific cohort. Responses to the specific interview questions (about satisfaction with the treatment, support received, helpful and less helpful aspects of the programme, helpful and less helpful therapist actions, maintaining motivation and actions to ease participation) were initially analysed separately and coded into topics. Many of these topics overlapped significantly, supporting the identification of themes. Once themes were fully supported by quotes identified in the interviews, two team members co-analysed and reflexively discussed the validity of each theme to ensure credibility and methodological rigour of the analysis.

## 4 Results

### 4.1 Questionnaire

#### 4.1.1 Helpfulness of website features

The total mean of website feature helpfulness was 3.44 (sd = 1.39) with most features being rated with a 3 or above (Table 2). The most helpful features were the ‘Behavioural Experiment Log’ and ‘Written Case Examples’.

**Table 2:** Mean helpfulness of website features.

OSCA feature	Mean	SD
Video examples	3.59	1.41
Written case examples	3.76	1.34
Street surveys	3.71	1.57
Attention training exercises (video & audio)	3.65	0.99
Testimonies from previous participants	3.59	1.22
Behavioural experiment log	4.29	0.98
Webcam	2.47	1.50
My model	3.06	1.74
Behavioural experiments via the webcam	2.82	1.59

#### 4.1.2 Helpfulness of communication

All forms of communication were rated above 3, apart from ‘Webcam chats’ rated at 2.35, with total forms of communication mean = 3.66, sd =1.41 (Table 3).

**Table 3:** Mean helpfulness of method and content of communication.

Method and content of communication	Mean	SD
Messaging function (emails) within the website	3.53	1.62
Emails to your personal account	3.24	1.60
Automated SMS text messages	3.65	1.90
Personalised SMS text messages	4.53	0.71
Phone calls	4.71	0.58
Webcam chats	2.35	2.02
Suggestions for new behavioural experiments	4.59	0.71
Clarification of completed experiments	4.47	0.94
General encouragement	4.76	0.43
Helping me re-examine my beliefs	4.71	0.47
Explaining things in the program that weren't clear	4.56	0.72
Reminders (e.g., to log in, complete behavioural experiments)	4.44	0.72
Total mean	3.66	1.41

Participants were also given the opportunity to provide a free text response to the question “What kind of things did your therapist say that you found particularly helpful?”. Within the 17 responses, the most common topics were: clarifying completed behavioural experiments or suggestions for future experiments (*n* = 9, 53 %); positive reminders of how far they had progressed (*n* = 8, 47 %); understanding socially anxious thoughts or beliefs (n = 6, 35 %); praise and encouragement from the therapist (*n* = 6, 35 %). Most responses included more than one topic, for example, *“She helped guide what I was trying to say if I became stuck, helped me see the positive outcome of some experiments I did, was very supportive of things I managed to accomplish”*. One participant mentioned - *“It was useful to go through things from the modules with a real person and have things reinforced, not just from a computer.”*

#### 4.1.3 Frequency of contact

Fifteen participants rated their satisfaction as 3, which represented ‘*Just the right amount’*, with two participants rating a 4, indicating slightly more than the right amount. The mean score was 3.11 (sd = 0.33).

### 4.2 Semi-structured interview

Thematic analysis found four themes: Satisfaction with online delivery of the treatment; Flexible communication supports encouragement to remain motivated; Helpful content of the treatment programme; Challenges.

#### 4.2.1 Satisfaction & support ratings

The mean satisfaction rating was 4.24. Seven participants rated their satisfaction at 5, nine participants rated their satisfaction at 4, while another participant rated their satisfaction at a 4.5. The mean rating of how supported participants felt during treatment was 4.94. Sixteen participants rated their support as 5, while one rated their support as 4.

#### 4.2.2 Satisfaction with online delivery of treatment

This theme reflects the satisfaction of being able to work on modules at one's own pace, at a time and place to best fit with other commitments. Online access allowed young people frequent recap of the content of the modules and of their own responses and contributed to the sense of being supported through therapy.

*“I think I liked the fact that the treatment is online and I can always go back to it. And if I was having worries, I could always go back to it and I know it's always there, and I can log on and reassure myself. So, I kind of like that I can access it from lots of different places”*

(P6)

*“Um well I could just text her any time if I felt there was a problem with how I was feeling or with the online and I could just log on to the website any time, so yeah that would be very supported."*

(P1)

Although the main content of the programme was online, the weekly telephone calls with the therapist guiding the treatment were an important contributor to participants' satisfaction.

The calls helped clarify the modules, pulling out key points, recapping on the young person's responses to the module, as well as selecting the most appropriate module to be released next. The call could be closely focused on the highly individual presentation of the person's problem because the therapist could read the young person's responses and look at their questionnaires before the call.

*“I think the weekly calls kind of motivated me to make sure I was on top of things. I think it was really like helpful for me, cause I was kind of getting stuck in my own head and it's difficult”*

(P17)

It was notable that participants commented on the helpfulness of the breadth and variety of aspects of the online programme, which are especially easy to access from within the same platform. These included street surveys, videos of other people describing their experiences of the treatment and virtual audiences. Although the content can be made available as part of face-to-face therapy, such ready access is a specific feature of the internet version of treatment and may be especially important for adolescents.

#### 4.2.3 Flexible communication supports encouragement to remain motivated

Methods for the participant to contact the therapist between weekly calls and for the therapist to send messages and reminders are built into the treatment programme. Participants commented on the flexible nature of communication. They valued the opportunity to be able to contact their therapist at any time via the messaging functions. This was seen as a benefit to support and maintain progress and was a common feature of satisfaction with the treatment. The choice of how to receive alerts or reminders (via SMS or email) was appreciated, as was flexibility about the frequency of communication.

*“I think being able to call the therapist... to talk about like your problems is really satisfying … like it reassured you that like at any stage you're able to contact them or send them a message through the online website which is good.”*

(P2)

*“I knew I could just message her and say this is a problem or if I needed to kind of talk through an experiment or a module, I could just message her… the messaging function was really helpful”*

(P3)

In some instances, reminders from the therapist to complete regular spontaneous experiments, as well as planned experiments, ensured that specific fears and worries were worked on regularly, contributing to overall progress.

*“Reminders to do the experiment were very helpful… I didn't go on the website too often and so I'd forget that I had an experiment and if I remembered then I would forget what experiment it was. So sometimes my therapist would send text messages to remind me to do certain experiments or she would remind me to put a SMS reminder on the experiment log”*

(P12)

Young people were also encouraged to use the reminder function within the treatment program to schedule SMS messages for their planned activities throughout the week, for example when to do behavioural experiments.

Specifically, encouragement by the therapist was a key factor in participants' satisfaction with the treatment and increased their motivation to continue with treatment, and plan and carry out behavioural experiments. This was enhanced by the flexible communication as small regular reminders between weekly phone calls were reported as helpful. Participants noted that the therapist offered encouragement when they were either hesitant, scared, or lacked the motivation to continue with treatment or their experiments.

*“um she's very encouraging, she helped me a lot and she also talked through things with me and helped me to do exercises, sorry experiments, that I wouldn't want to do. And like every week or so she would say ‘oh have you done it’ and then message like ‘make sure like you do the experiments’. She was being very motivating, encouraging and that mainly helped me through”*

(P2)

*“I think just like, providing encouragement, like constantly, and also just making sure that I kind of understood and what the aims were, like, each week. So the weekly phone calls were quite like, helpful with just having like a routine to stick to”*

(P4)

Although not referred to in these terms, the impression of therapy being a joint endeavour between participant and therapist was strong.

*“And anytime I accomplished something that I had already said I found difficult, she'd be very supportive and help me towards it. And when I did face it, she'd celebrate with me”*

(P12)

Therapist-specific factors, namely, the therapist's ‘friendliness’ as well as a ‘supporting’ and ‘reassuring’ approach during participant-therapist interactions were an important factor in how well supported participants felt.

#### 4.2.4 Helpful content of the treatment programme

In addition to the quantitative data described previously, where they were asked to rate the helpfulness of each module, during the interview participants were asked if any aspects of the programme content were particularly helpful.

*“um I think I think it was all helpful in like different ways. I can't think of anything. I can't pinpoint one thing that was unhelpful.”*

(P5)

No aspects of content were described as unhelpful, although some were viewed as challenging, as explained in theme of Challenges.

“I don't think that (there) was anything, like, that wasn't helpful, I thought all of it was quite helpful for me,… just because it made me feel a little bit uncomfortable was when we did the webcam and I had to watch those videos back. I got, like, really uncomfortable doing that - that's part of my anxiety - however that was there to help me and it actually did.”

(P2)

Key components of treatment include learning about feeling self-conscious and training in externally focused attention.

*“The (self-conscious) module was helpful because it, like, explained, like um, it explained that people don't see how you feel and then I think it gave how you can do behavioural experiments to test it, which was helpful a lot. Because obviously being able to test it is beneficial, the most beneficial.”*

(P8)

“I think the best thing was that the getting out of my head one, the one that has exercises like the sounds and the colours… well, it did its job cause it got me out of my head and also, like, I could go back to it and use it again and it was like an effective exercise basically”

(P15)

Another key component of therapy, behavioural experiments, is described in several core and optional modules. Participants saw behavioural experiments as an important part of therapy.

*“I think doing the behavioural experiments was very helpful because it kind of put all of the, like, theory into practice and it made me see, like, the benefits because it made me, like, do the activities which was, like, talking about, like, in the modules”*

(P4)

Some modules for specific issues such as sweating, blushing, or feeling boring are only released if the therapist and young person decide these would be helpful. Where applicable to the participant, these modules were mentioned as having been helpful.

“The blushing one - I just feel like that really helped me because it was one of my main fears, so it just really, like, helped me to realise that people don't really see it”

(P13)

‘Managing my inner critic’, another of the optional modules that was released to many participants was described as helpful. The module enabled participants to understand the unhelpful effects of self-criticism, to notice when it occurred and shift to a kinder mode of self-talk.

*“okay I'd say in particular the module on managing my inner critic (was helpful) cause I tend to see myself quite negatively. So that kind of helped me be more kind to myself”*

(P11)

Learning about specific issues was also strengthened by being able to watch street surveys, which gather together a range of views on a particular topic such as sweating or making mistakes. Passers-by on the street were interviewed whilst being recorded. Participants noted this was helpful because they were able to see things from other people's perspective and learned that their fears were not viewed as negatively as they thought.

*“I liked the street surveys, like, seeing things from other people's perspectives and, like, how other people view all these things I always viewed in a negative way. Like blushing or anything like that, and see how other people reacted differently”*

(P5)

Other features accessible to all throughout their treatment such as case examples, or viewing and reading about the experiences of people who had previously completed treatment, were described as helpful. Some participants noted that they would prefer if more case examples were of a similar age to themselves.

“Reading examples … or, like, this watching video of people who had gone through this, like, people who had done the treatment”

(P10)

#### 4.2.5 Challenges

This theme focused on: the amount of content; challenging but helpful experiments; and practical considerations.

Some participants experienced difficulty staying motivated during treatment, which they all attributed to the amount of reading and writing they were required to do on the program each week. For example:

*“Sometimes I felt like, oh it's going to be so long, like, there's a lot of reading to do, just because my attention span is quite short”*

(P2)

*“Um I don't have any problems staying motivated but sometimes I did struggle to sort of do everything I had to do for the next phone call because obviously schoolwork and trying to do everything especially if there were any behavioural experiments before the next phone call and then put it all int the experiments book was kind of tricky sometimes”*

(P8)

Completing weekly questionnaire packs was sometimes seen as difficult and time-consuming. On the other hand, for some young people, being able to see the questionnaire measures as a graph served as a visual reminder of progress.

*“I also thought having a questionnaire to do like every time was cool … and I thought that was really useful because then you can track on the graphs your, how good you're getting in, like, less anxious …. provides encouragement and motivation continues because it shows your improvement and looks like it's gradually getting better”*

(P2)

Those participants who had not experienced difficulties in staying motivated and engaged used techniques such as reminding themselves about why they were participating in the treatment, their progress so far and their hopes for a better outcome. This may partly explain why regular encouragement from the therapist was also such an important factor in engagement, as described in a previous theme.

*“It was just the fact that I knew like when I got to the end, I'd be proud of myself and that ultimately it would help me so I know it was something that was going to improve my life really”*

(P13)

Although no aspects of treatment were described as unhelpful, when asked further, participants did report that they found some aspects challenging. Specifically, they identified facing a fearful situation, initially in the form of meeting and speaking with the therapist and later in the form of behavioural experiments, as a difficult aspect of treatment. Importantly, they also clearly stated that this was the most helpful part of treatment.

*“The first time I did it, it was really nerve wracking, but again it was helpful in the long run if you want to speak to people. But at the time it was, yeah, I didn't quite enjoy it. I was quite dreading to do it. But yeah it was helpful”*

(P1)

Some practical aspects of participating were also described as challenging. When phone calls were scheduled during school lunch breaks or study periods it was sometimes difficult to find an appropriate space in school to have the telephone call. Participants noted that this could lead to wasted time looking for a room or being allocated a quiet, private room for their call. The inconvenience of calls during school time was also cited as an issue.

## 5 Discussion

This mixed-methods study examined participant satisfaction and overall experience of a therapist-assisted internet-delivered cognitive therapy adapted for adolescents with SAD (OSCA). After receiving OSCA, participants completed a treatment acceptability questionnaire and took part in a semi-structured interview. Overall, results suggest that OSCA is an appropriate and acceptable treatment for adolescents with SAD.

Helpfulness and treatment acceptability were rated as high. Participants rated similar aspects of OSCA especially helpful, in particular behavioural experiments and training in an external focus of attention, both of which are key elements of the treatment programme. In addition, optional modules that were released to meet a young person's specific needs received high ratings of helpfulness and featured strongly in the interview data. The finding points to the value of online treatments that can be personalised to a young person's particular needs. Interestingly, the aspects of treatment that some participants reported finding difficult were the same elements of therapy rated as most helpful. For example, participants mentioned that behavioural experiments, such as wearing blusher to learn about how other people respond, were uncomfortable and anxiety-provoking. However, participants reported that these challenges were the most powerful. This explains the lower average rating given to ‘Behavioural Experiments via the webcam’ on the questionnaire; when asked further during the interview, participants reported that they had rated these specific behavioural experiments as less helpful because they were challenging and uncomfortable, but they recognised that they were an important part of tackling their social anxiety and had contributed to their symptom improvement. These findings are consistent with the themes in qualitative studies of adolescents treated with face-to-face Cognitive Therapy (Taylor et al., 2021) and with iCBT (Smart et al., 2023).

The most helpful communication methods were phone calls and personalised SMS text messages, demonstrating that the variety of methods of communication available through the programme is appreciated. Participants rated all aspects of the content of communication very highly. Participants also thought communication frequency was “just…right”. Such variety, frequency and ease of communication is an in-built feature of the online treatment programme but is difficult to achieve with face-to-face therapy. Free text responses indicated that participants viewed help from the therapist as important to progress with key components of the treatment, such as behavioural experiments and altered cognitions, and that they were supported in this by therapist guidance, praise, and encouragement.

The online nature of the therapy was appreciated by many participants. Convenience and flexibility of access to treatment modules and supporting materials as well as the ability to easily recap on previous material were reported as helpful but participants were also clear that the guidance and encouragement of the therapist was a key factor. Online delivery seemed to enhance the support participants felt throughout therapy, as it allowed them to communicate with their therapist in a flexible, effective and efficient manner via the messaging function and phone calls. This was further enhanced by the therapist's approach and the encouragement participants often received through the website messaging function. The positive feedback from participants about the online nature of OSCA treatment is interesting to consider in light of studies reporting a preference amongst adolescents for face-to-face delivered treatment, with only around 1 in 6 teens expressing a preference for online delivery (Hollis et al., 2017). It has been suggested that this is because of concerns about less therapist contact and a lack of individual tailoring of digital treatments (Hollis et al., 2017; Wisman et al., 2023). It is therefore possible that the amount and nature of therapist assistance as well as the relatively high level of personalisation with OSCA contributed to the high acceptability we observed. Future studies could examine this further.

This study also gathered information about preferences for the timing and venue of online treatment. Some participants had their therapy calls during school hours. Unfortunately, some experienced delays and reported additional stress due to lack of an appropriate room to accept the calls. It is important that future research and therapy consider whether schools can offer a designated space that participants can use for calls with their therapists. Other participants completed most of their therapy sessions at home due to COVID-19 restrictions and school closures. While some participants were comfortable with taking calls at home, some participants reported that they felt uncomfortable due to their family being at home and had concerns about being heard. There are many benefits to delivering therapy outside a clinic in locations such as home and school, but there are also important challenges such as ensuring confidential space, that need to be considered. An interesting point raised by Smart et al. (2023) in their qualitative study of adolescents who had received iCBT for mixed anxiety disorders (BRAVE-online) was that whilst adults may appreciate the flexibility afforded by internet treatments, adolescents may find it more burdensome finding time to complete treatment around their school hours and activities.

A challenge for ten participants was difficulty in staying motivated. This should be taken into consideration for future studies as some participants may be dissuaded from completing treatment due to a lack of motivation. However, it is worth noting that of the 22 participants randomised to the OSCA treatment arm, only two dropped out, suggesting that even though some found engagement challenging they still persisted with and benefited from treatment (Leigh and Clark, 2023). It was helpful to learn from the seven participants who reported no problems in staying motivated that they were helped by reminding themselves that the treatment would help therapist encouragement and reminders. Specific aspects of the online programme that support this encouragement are: seeing graphs of treatment outcomes for other adolescents, watching videoclips of young people talking about the benefits they derived from the treatment programme, reviewing graphs of their weekly questionnaire scores to observe their own improvement. The importance of therapist reminders and encouragement, and use of the in-built reminder systems in the programme should be emphasised when training clinicians in the delivery of OSCA.

Findings from this study are in accordance with what we expected, high ratings of helpfulness and high treatment acceptability. These results are also supported by previous findings in adult populations treated using online therapy. Halmetoja et al. (2014) examined participants' experience of iCBT in adults for anxiety disorders, four years post treatment. Their results showed that all participants reported the treatment as having some effect, but it was also difficult and demanding, while a vast majority of participants appreciated hearing about previous participants' experiences. Furthermore, all participants acknowledged the helpfulness of exposure modules, which are similar to the behavioural experiments used in OSCA, a finding mirrored in a qualitative study of iCBT for anxiety in adolescents (Smart et al., 2023). Similarly, Asplund et al. (2019) studied the experiences of adults with anxiety who underwent iCBT. They found that many participants reported positive effects of the treatment, in daily life and their mental health; while they also reported the importance of the therapist support they received, which was attributed to the online nature of the therapy.

There are a number of strengths of this study. Two types of data collection were used: an online treatment acceptability questionnaire and a semi-structured interview. The semi-structured interview allowed participants to take their time to open up about sensitive issues and to provide as much information as possible. Gathering both types of data was to maximize the learning about the views and experiences of this initial cohort who had completed treatment using OSCA and to identify the themes, which further increased our understanding of positive and negative aspects of treatment. The interviews took place shortly after participants had finished treatment to reduce issues associated with memory and to ensure more accurate answers.

The small sample of 17 participants were Black British, White British and Mixed Ethnic Group British. All were female which limits the generalisation of the findings. It should also be considered whether the current study population can be compared to a clinic referred population since the participants recruited through schools. As all participants had experienced SAD there is a possibility that speaking with a stranger may have been stressful and therefore influenced their interview responses, especially those interviewed using video conferencing. However, this is balanced by not having a potential conflict of interest if interviewed by the therapist with whom they had formed a therapeutic alliance. Feedback on the programme was only requested at the end of treatment, when there had been a significant reduction in symptoms so does not necessarily reflect how the participants felt during treatment. Three participants (all female also) did not take part in the interviews. Two of those had slightly higher social anxiety scores at the end of treatment compared to those who agreed to participate in the interview, therefore the sample is not fully representative of the range of treatment outcomes. Succeeding in interviewing these participants could have offered some additional insight towards obstacles, or opportunities, to further improve OSCA. We note the lack of males in our sample. There was also a predominance of females (91 %) in the full RCT sample, despite recruiting from mixed schools. As a result, we do not know how well the treatment can be applied to male teenagers and whether there are similar or different issues around acceptability. This would be valuable to examine in a future study.

## 6 Conclusions

The results show promising evidence of the acceptability of an adolescent version of therapist-assisted online treatment for SAD. As with any novel treatment, acceptability as well as effectiveness is important. Participants' responses regarding treatment were overall very positive whilst no participant reported any major dissatisfaction or problems. The main disadvantage reported by a number of participants is that modules can sometimes be lengthy due to the amount of reading and writing. Participants found it helpful that they could access the treatment at any time and go through the therapy at their own pace. The ability to contact their therapist via the web programme was reported as very helpful, as was the encouragement and support received in messages from the therapist. It might be that these functions mitigated any potential concerns about reduced contact time with the therapist, but we note that because only one therapist delivered OSCA in the trial the relative effect of therapist to the outcome will likely be greater. Therefore, replicating this finding across different therapists will be an important next step.

This study, together with the results of the treatment trial of which these participants are a sub-sample (Leigh and Clark, 2023), highlights that OSCA could be a viable treatment option for adolescents with social anxiety. The therapy targets the adolescent population, which is the most sensitive time for the emergence of SAD. Since this therapy can be accessed irrespective of the location of the patient, it has the potential to be more readily available to those who need it. The rich learning points from this study will help inform future studies to improve access to treament.

## Funding information

Eleanor Leigh was funded by a Wellcome Trust Clinical Research Training Fellowship (102176/Z/13/Z) and is now funded by a MRC Clinician Scientist Fellowship (MR/W02389X/1). David M Clark was funded by the 10.13039/100010269Wellcome Trust (WT069777).The study was sponsored by the 10.13039/501100000769University of Oxford. The study was supported by the NIHR Oxford Health Biomedical Research Centre.

## Declaration of competing interest

None to declare.

## Appendix A Supplementary data

Supplementary material Image 1
