# Supplementary material for: Adolescents' experience of receiving internet-delivered cognitive therapy for social anxiety disorder
Source: Internet Interv. 2023 Sep 7;34:100664. doi: 10.1016/j.invent.2023.100664 (PMC10502334; doi:10.1016/j.invent.2023.100664)
Supplement: Supplementary file 1 — Supplementary material [file mmc1.docx]

**Supplementary Information**

**Self-report questionnaire items developed for the study**

**OSCA web features**

| For each feature, please rate how helpful it was to you in overcoming social anxiety: | | | | | | |
| --- | --- | --- | --- | --- | --- | --- |
|  | 0  (not helpful at all) | 1 | 2 | 3 | 4 | 5  (extremely helpful) |
| Video examples |  |  |  |  |  |  |
| Written case examples |  |  |  |  |  |  |
| Street surveys |  |  |  |  |  |  |
| Attention training exercises |  |  |  |  |  |  |
| Testimonies from other young people |  |  |  |  |  |  |
| Behavioural experiment log |  |  |  |  |  |  |
| Webcam |  |  |  |  |  |  |
| My model |  |  |  |  |  |  |
| Behavioural experiments via the webcam |  |  |  |  |  |  |

**Communication modes:** Please reflect on the ways your therapist communicated with you over the course of treatment.

| For each mode of communication, please rate how helpful it was to you: | | | | | | |
| --- | --- | --- | --- | --- | --- | --- |
|  | 0  (not helpful at all) | 1 | 2 | 3 | 4 | 5  (extremely helpful) |
| Messaging function (emails) within the website |  |  |  |  |  |  |
| Emails to your personal account |  |  |  |  |  |  |
| Automated SMS text messages |  |  |  |  |  |  |
| Personalised SMS text messages |  |  |  |  |  |  |
| Phone calls |  |  |  |  |  |  |
| Webcam chats |  |  |  |  |  |  |

**Communication content:** Please reflect on the content of your therapist's communication with you over the course of treatment.

| For each type of communication, please rate how helpful it was to you: | | | | | | |
| --- | --- | --- | --- | --- | --- | --- |
|  | 0  (not helpful at all) | 1 | 2 | 3 | 4 | 5  (extremely helpful) |
| Suggestions for new behavioural experiments |  |  |  |  |  |  |
| Clarification for completed experiments |  |  |  |  |  |  |
| General encouragement |  |  |  |  |  |  |
| Helping me re-examine my beliefs |  |  |  |  |  |  |
| Explaining things in the programme that weren’t clear |  |  |  |  |  |  |
| Reminders (e.g., to logon, complete questionnaires, complete behavioural experiments) |  |  |  |  |  |  |

**Communication frequency:** Please think about how satisfied you were with how frequently you and your therapist were in touch and rate below:

| 0  (not satisfied at all) | 1 | 2 | 3 | 4 | 5  (extremely satisfied) |
| --- | --- | --- | --- | --- | --- |
|  |  |  |  |  |  |

**OSCA Modules**

| Core Modules (allocated to all users) |
| --- |
| Introducing the treatment |
| Getting started |
| Feeling self-conscious |
| Safety behaviours |
| Attention & safety behaviours experiment |
| Watching your conversation videos |
| Getting out of your head and into the world |
| Behavioural experiments |
| Additional Modules (for particular fearful concerns and problems) |
| Blushing |
| Shaking |
| Sweating |
| Having conversations |
| Feeling boring |
| Feeling stupid |
| Feeling responsible for others enjoyment |
| Decatastrophizing |
| Worrying in advance |
| Going over social situations after they’ve happened |
| Leaving the past behind |
| Managing my inner critic |
| Self-esteem |
| Managing my mood |
| Giving myself credit |
| My therapy blueprint |
| \| Modules for to help patients prepare for follow-up sessions \| \| --- \| |
| Preparing for first follow-up |
| Preparing for second follow-up |
| Preparing for third follow-up |
